# Neurocognitive and brain structure correlates of reading and television habits in early adolescence

**DOI:** 10.1038/s41598-025-88398-2

**Published:** 2025-02-20

**Authors:** Andreas M. Rauschecker, Pierre Nedelec, Simon Pan, Maria Olaru, Ryan M. Nillo, Clare E. Palmer, Diliana Pecheva, Anders M. Dale, Terry L. Jernigan, Leo P. Sugrue

**Affiliations:** 1 Department of Radiology and Biomedical Imaging, University of California, San Francisco, San Francisco, CA USA; 2 Center for Human Development, University of California, San Diego, La Jolla, CA USA; 3 Center for Multimodal Imaging and Genetics, San Diego School of Medicine, University of California, La Jolla, CA USA; 4 Department of Radiology, San Diego School of Medicine, University of California, La Jolla, CA USA; 5 Department of Cognitive Science, University of California, San Diego, La Jolla, CA USA; 6 Department of Neuroscience, San Diego School of Medicine, University of California, La Jolla, CA USA; 7 Department of Psychiatry, San Diego School of Medicine, University of California, La Jolla, CA USA; 8 Department of Psychiatry and Behavioral Science, University of California, San Francisco, San Francisco, USA

**Keywords:** Brain MRI, Imaging, Brain development, Neurocognition, Behavior, Neuroscience, Cognitive neuroscience, Intelligence, Reading, Brain

## Abstract

Results of the impact of reading books and viewing television on neurodevelopment have been mixed, without definitive evaluation to date. Using data from 11,875 US adolescents in the Adolescent Brain and Cognitive Development (ABCD) study, we investigated the associations between reading and television viewing on brain morphology and neurocognitive performance. After quality control, 8,125 participants’ MRI scans and cognitive tests were analyzed in relation to their reading and TV habits. Greater reading time was associated with higher cognitive performance and regionally-selective increases in cortical area, while greater TV viewing had a much smaller association with lower cognitive performance and decreased cortical area. Regionally, areas of spatial overlap in associations included the lateral temporal, inferior parietal, and inferior frontal lobes, while significant associations in the ventral and inferior temporal cortex and cingulate cortex were unique to reading habits. These relationships persisted after adjusting for demographics, socioeconomic factors, genetic ancestry, and imaging factors. The magnitude of reading associations exceeded those of TV viewing and was similar to established contributions of parental income and education on neurodevelopment. This study provides a comprehensive evaluation of how these behaviors correlate with early adolescent brain development across a large diverse population.

**Supplementary Information:**

The online version contains supplementary material available at 10.1038/s41598-025-88398-2.

## Introduction

Early adolescence represents a period of rapid brain and cognitive development during which a wide variety of environmental and behavioral factors have the potential to affect neurocognitive outcomes^1^. Among the myriad ways in which children and early adolescents spend their time, reading and television viewing are two common recreational activities that are generally regarded as having opposing positive and negative associations with brain health, despite a lack of conclusive evidence across large populations.

The American Academy of Pediatrics has issued statements and guidelines regarding possible health effects of television viewing^2^, based on literature that suggests a negative association of screen time and television viewing on neurocognitive development^3–5^. Conversely, reading is generally perceived as a cognitively beneficial habit, and neural correlates of reading (and learning to read) have been well-documented^6–8^. Indeed, there is evidence that screen time and reading have opposing and spatially different effects on functional connectivity patterns in the adolescent brain^9^. However, the neurocognitive and neurostructural effects of television viewing and reading during childhood/early adolescence have not been systematically investigated on a large scale in a demographically diverse population. Determining the precise associations of these behaviors on brain structure and neurodevelopmental outcomes is of significant neuroscientific interest and of public health concern, particularly as highlighted by the COVID-19 pandemic, during which overall screen time increased substantially for children and adolescents^10^.

To better understand the associations between common youth behaviors and neurocognitive development, large population-based studies are needed^11^ to reliably estimate effects and control for confounding factors. The Adolescent Brain Cognitive Development (ABCD) study^12,13^ is an ongoing longitudinal study of a large demographically diverse cohort of developing adolescents, aged 9–11 years at the time of enrollment and recruited from 21 sites across the United States, that is unprecedented in its magnitude, diversity, extent of data gathered, and harmonization of protocols across acquisition sites. Participants undergo serial multimodal brain imaging, including high-resolution structural MRI, genetic sampling, and psychosocial surveys with the overarching goal of assessing the genetic and environmental factors that contribute to neurodevelopment and brain health.

In the current study, we leveraged this large dataset to determine the associations between daily reading and television viewing, cognitive performance, and brain structure while controlling for potentially confounding associations such as socioeconomic status, parental education, genetic ancestry, and other demographic factors. The hypotheses being tested are that reading and television viewing are associated with higher and lower cognitive performance, respectively, and with opposing morphological associations in the developing brain.

## Methods

### ABCD study and participants

The ABCD study is a longitudinal cohort study consisting of 11,875 children aged 9–11 at study onset between September 1, 2016, and November 15, 2018. Participants were recruited through the school system across 21 study sites in major metropolitan areas across the United States^13^. Most ABCD research sites rely on a central Institutional Review Board (IRB) at the University of California, San Diego, for the ethical review and approval of the research protocol, with a few sites obtaining local IRB approval. Written informed parental/guardian consent and child assent were obtained from all participants. All data collection, data storage, and analyses were performed in accordance with IRB guidelines and regulations. Serial MRI, bio-sample collection, neurocognitive testing, and psychiatric, social, and behavioral questionnaires are administered on an annual or bi-annual basis. The current study is based on imaging, test results, and survey data from participants included in the National Institutes of Mental Health Data Archive (NDA) public release of the baseline data from all children enrolled in the ABCD study (NDA Release 3.0). All data collection, data storage, and analyses were performed in accordance with IRB guidelines and regulations.

### Exclusion criteria

Within the ABCD study, any child aged 9–11 within the regular school system was eligible for enrollment, with exclusionary diagnoses including a current diagnosis of schizophrenia, autism spectrum disorder (moderate, severe), mental retardation/intellectual disability, or alcohol/substance use disorder. Of note, a diagnosis of dyslexia was not recorded and was not an exclusionary criterion for ABCD or for our study. The baseline population of 11,875 children were filtered based on imaging quality control, missing data, and outlier exclusion for behavioral measures of interest. Of the 11,875 participants enrolled in the study, 65 did not complete MRI, resulting in a total of 11,810 participants with structural neuroimaging data. Each MRI dataset is further assessed for technical data quality (e.g., excessive motion) and undergoes radiologist review for major anatomical abnormalities. 10,783 participants receiving a passing score for these quality indicators were included in our analysis. Participants missing data about number of reading hours or TV viewing hours were excluded (*N* = 766). For the measure of daily hours spent on pleasurable reading, a subset of participants reported unrealistic values that far exceeded the mean time. Therefore, we excluded participants reporting > 8 h/day of pleasurable reading (*N* = 49) and any participants with missing behavioral, imaging, or demographic data, resulting in a total of 8,125 participants in the final analysis.

### Behavioral questionnaires

Daily hours spent reading for pleasure or television viewing were derived from parental questionnaires and coded as pseudo-continuous variables. For reading, participants’ caregivers were asked to report the number of hours per week that their child engaged in pleasurable reading. For consistency with daily television viewing, this number was divided by seven to convert it to daily hours spent reading. Participants reporting daily reading exceeding 8 h were filtered as outliers as described above. For television viewing behavior, participants were asked in a survey to indicate from seven potential answers (“None”, “< 30 minutes”, “30 minutes”, “1 hour”, “2 hours”, “3 hours”, and “4 + hours”) how much time they spent viewing television shows or movies on a typical weekday and on a typical weekend day. These seven categories were transformed to the corresponding numerical values (0, 0.25, 0.5, 1, 2, 3, 4) and a weighted average across weekday and weekend responses was computed for regression as a pseudo-continuous variable. For the purposes of visualization, the first three values were combined into one category (i.e., 30 min or less).

### Neurocognitive assessments

Neurocognitive performance was assessed using the well-validated NIH Toolbox Cognition Battery^14^ that evaluates neurocognitive performance using seven different sub-tests covering cognitive domains such as language, executive function, working/episodic memory, and processing speed. The toolbox also includes three composite scores: (1) fluid cognition, which is thought to represent reasoning and problem-solving ability independently of acquired knowledge; (2) crystallized cognition, which is thought to represent knowledge, including language, acquired from prior learning and past experiences^15^; and (3) a total composite score. Raw (non-age-corrected) standard scores for each sub-test and composite score were extracted from the baseline ABCD dataset. NIH Toolbox standard scores have a normative mean of 100 and standard deviation of 15 and compare the performance of the test-taker to those in the entire NIH Toolbox nationally representative normative sample, regardless of age or any other variable. Given the demographic diversity in the sample, all statistical analyses controlled for age, sex, ethnicity, genetic ancestry, family ID/education/income/marital status, and geographical testing site. As reading and television viewing times were demonstrated to be anti-correlated, analyses using the independent variable of reading included television viewing as a fixed effect, and vice versa. Effect sizes of individual factors were estimated by calculating the difference in variance (R^2^) explained by a ‘full’ model that included all covariates compared to a ‘reduced’ model that lacked the factor of interest (https://CRAN.R-project.org/package=MuMIn, version 1.46.0).

### Magnetic resonance imaging and image processing

The MRI acquisition protocols and centralized image processing steps employed in the ABCD study have been described in detail elsewhere^12,13^. Briefly, MRI protocols were harmonized across scanner platforms at the 21 enrollment sites, all imaging data were obtained on 3T scanners using either 32-channel head or 64-channel head/neck coils, and image consistency was maintained through regular quality control using both a standard mechanical and traveling human phantom. The Data Analysis, Informatics, and Resource Center within the ABCD Study was responsible for performing quality control assessment for motion artifact, intensity inhomogeneity, white matter underestimation, pial overestimation, magnetic susceptibility artifact, and incidental findings.

Brain structure was assessed through cortical morphology measured on a vertexwise and atlas-based region-of-interest (ROI) basis. Cortical surface reconstruction was performed via FreeSurfer 5.3.0 (https://surfer.nmr.mgh.harvard.edu) using T1-weighted images with parcellation of cortical ROIs according to the Desikan-Killiany Atlas^16^. These analyses are explained in further detail as follows.

### Region of interest (ROI) analyses

Baseline (year-1) data from the NDA RDS 3.0 release of the ABCD dataset was used as the source of the ROI data. For each ROI in the Desikan-Killiany Atlas, average cortical volumes and areas were extracted from the NDA dataframe and were consecutively set as the dependent variable in a generalized additive mixed model (https://cran.r-project.org/web/packages/gamm4/index.html, version 0.2-6). Given the demographic diversity in the sample, all statistical analyses controlled for age, sex, ethnicity, genetic ancestry, family ID/education/income/marital status, MRI device serial number, and MRI software version. Similar to neurocognitive performance analyses, because reading and television viewing times were anti-correlated, analyses using the independent variable of reading included television watching as a fixed effect, and vice versa. P-values were corrected for multiple comparisons using the Benjamini and Hochberg false discovery rate (FDR) procedure^17^ at an alpha level of 0.05 (https://CRAN.R-project.org/package=sgof, version 2.3.2). As with neurocognitive assessments, effect sizes of individual factors on ROI based morphological measures were estimated by calculating the difference in variance (R^2^) explained by a ‘full’ model that included all covariates compared to a ‘reduced’ model that lacked the factor of interest.

### Vertexwise analyses

To provide a higher spatial resolution whole-brain analysis that is not biased by the arbitrary borders and volume averaging problems inherent in an ROI based approach, we also performed analyses at the vertex level. Vertexwise cortical area and thickness were set as the dependent variables in linear mixed models using the fast and efficient mixed-effects algorithm (FEMA) described in detail elsewhere^18,19^. This algorithm models linear effects while controlling for confounding variables and the random effects of participant and family relatedness in a computationally-efficient way across 20,484 gray matter locations, or vertices. Vertexwise statistical analyses controlled for the same fixed and random effects as ROI analyses. The vertexwise data were computed from ABCD 4.0 imaging data because of missing data in the 3.0 release. Nonetheless, 5 individuals did not have vertexwise data available for analysis. Due to covariance across the cortex the number of independent tests is much smaller than assumed by a conservative mass-univariate correction for multiple comparisons, such as the Bonferroni method. Therefore, to assess significance of whole-brain vertexwise results we applied the Multivariate Omnibus Statistical Test (MOSTest)^20,21^, a less conservative estimate that accounts for spatial covariance in vertexwise cortical morphology across the brain, with significance computed by running 10,000 wild bootstrap permutations.

## Results

In the current study, we analyzed 8,125 participants (mean age 9.93y, 3,854 female, Table 1) out of the 11,875 in the ABCD study 3.0 data release after filtering for imaging quality control, missing data, and behavioral outliers. The mean daily hours of pleasurable reading was reported as 0.60, with a standard deviation (SD) of 0.72. The mean daily hours of television viewing was reported as 1.09 [SD 1.07]. Daily hours of pleasurable reading and television viewing were positively and negatively associated, respectively, with sociodemographic factors such as parental income and education (eFigure 1 in the Supplement), highlighting the importance of including these variables as confounding factors in mixed-effects analyses. Moreover, daily hours spent reading was slightly inversely associated with daily television viewing across participants (mean β ± SEM = −0.29 ± 0.048, t-stat = −6.11, p-value = 1.06 × 10^−9^) (eFigure 2 in the Supplement). Therefore, all mixed effects analyses of reading included TV viewing as a fixed effect, and vice versa, to isolate the independent associations of each behavior.

**Table 1:** Demographics of study participants.

		Total ABCD cohort No. (%)	Study subsample No. (%)
*n*		11,876	8125
Age in months, mean (SD)		118·98 (7·50)	119·12 (7·50)
Sex, n (%)	Female	5680 (47·8)	3854 (47·4)
	Male	6196 (52·2)	4271 (52·6)
Race, n (%)	Asian	275 (2·3)	180 (2·2)
	Black	1869 (16·0)	1004 (12·5)
	Other/Mixed^(a)^	2037 (17·4)	1335 (16·6)
	White	7524 (64·3)	5526 (68·7)
Hispanic or Latino, n (%)	No	9312 (79·4)	6562 (80·8)
	Yes	2411 (20·6)	1563 (19·2)
Highest parental educational level, n (%)	Less than High School Diploma	593 (5·0)	278 (3·4)
	HS Diploma/GED	1132 (9·5)	593 (7·3)
	Some College	3079 (26·0)	1997 (24·6)
	Bachelor	3015 (25·4)	2224 (27·4)
	Post Graduate Degree	4043 (34·1)	3033 (37·3)
Total household income ($), n (%)	[< 50 K]	3223 (29·7)	2153 (26·5)
	[ > = 50 K & <100 K]	3071 (28·3)	2367 (29·1)
	[ > = 100 K]	4564 (42·0)	3605 (44·4)
Parents married, n (%)	No	3790 (32·2)	2319 (28·5)
	Yes	7990 (67·8)	5806 (71·5)
Daily time spent pleasurable reading, mean (SD)		0·66 (1·32)	0·60 (0·72)
Daily time spent television viewing, mean (SD)		1·12 (1·10)	1·09 (1·07)

^(a)^Includes American Indian, Alaskan Native, Native Hawaiian, other Pacific Islander, mixed, and not otherwise listed.

## Associations between reading/television viewing and neurocognitive performance

**Fig. 1:**
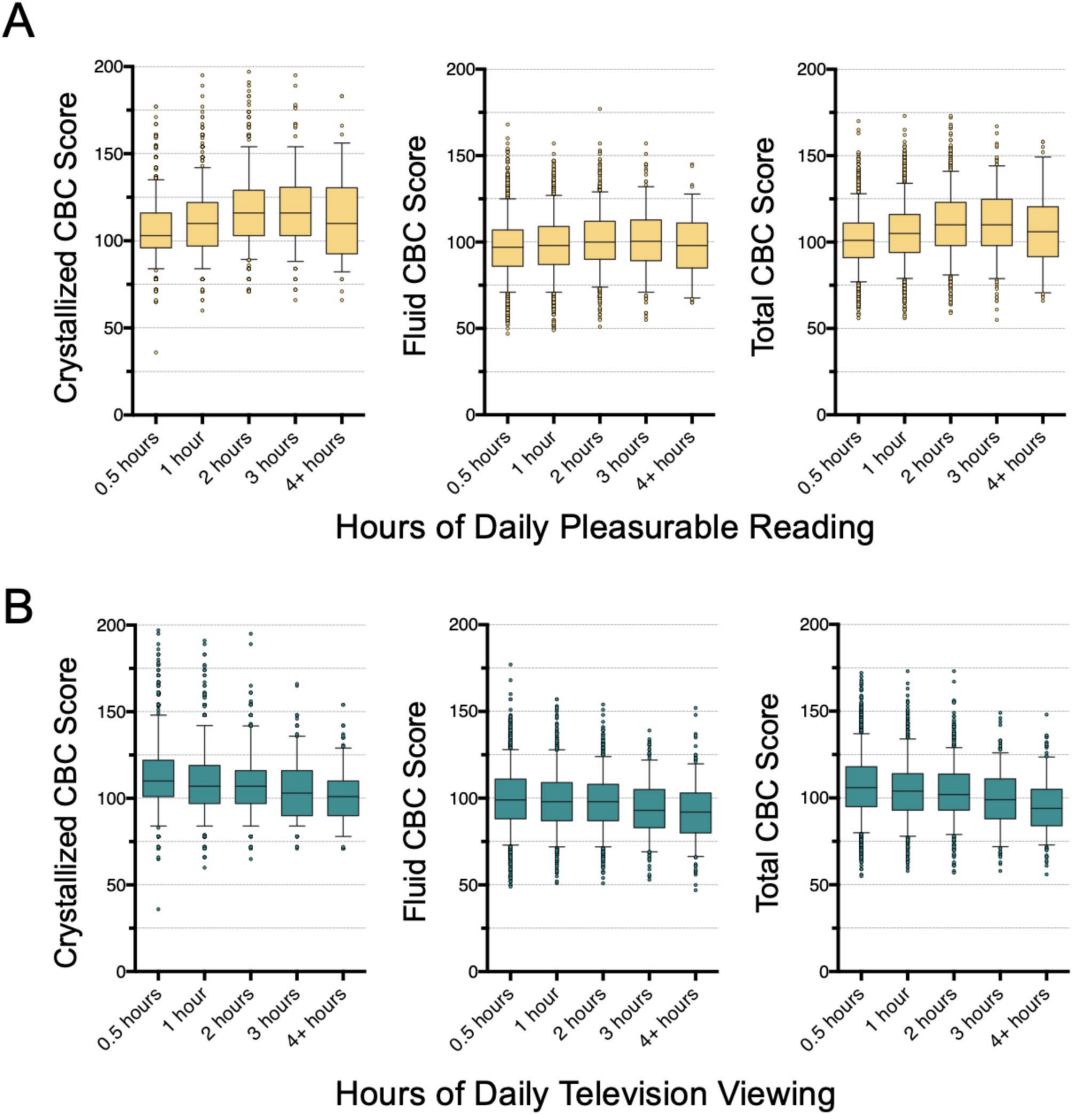
Associations between NIH Cognition Battery Composite (CBC) Scores and (**A**) Reading and (**B**) Television Viewing. Box and whisker plots depicting the raw (non age-corrected) standard composite scores from the NIH Cognition Battery test for each binning of reported hours spent pleasurable reading (top row, orange) or television viewing (bottom row, teal). Statistical results from linear mixed effects models for these data are summarized in Table 2. The center line denotes the median, the boxes demarcate the interquartile range, and the whiskers denote the 5th and 95th percentiles, with outliers represented as individual points.

Children in the baseline ABCD dataset were administered the NIH Toolbox Cognition Battery^22^ that assesses neurocognitive performance through seven different sub-tests covering cognitive domains such as language, executive function, working/episodic memory, and processing speed. The toolbox also includes three composite scores: (1) fluid cognition, which is thought to represent reasoning and problem-solving ability independently of acquired knowledge; (2) crystallized cognition, which is thought to represent knowledge, including language, acquired from prior learning and past experiences^15^; and (3) a total composite score. Generalized additive mixed models were constructed to determine the associations between daily pleasurable reading/television viewing and performance on the seven sub-tests and three composite metrics in the NIH Toolbox Cognition Battery. Daily reading was positively associated with all seven cognitive sub-tests and three composite scores (Fig. 1A; Table 2). Note that while the overall correlation was positive, at 4 or more hours of reading per day, there was no significant correlation between the three composite metrics and reading hours (eFigure 3 in the Supplement). Conversely, daily television viewing was negatively associated with all seven cognitive sub-tests and three composite scores (Fig. 1B; Table 2). Notably, reading was more strongly associated with performance on tests of crystallized cognition (effect size β = 2.45) than fluid cognition (β = 1.61), while television viewing was similarly associated with both composite metrics (β = −0.87 for crystallized cognition; β = −0.80 for fluid cognition). Overall, effect sizes per hour of activity were much larger for reading than for television viewing (Table 2). One hour of additional daily reading was associated with a 2.5-point increase in crystallized performance, and one hour of additional daily television viewing was associated with a 0.9-point decrease in crystallized performance, on a scale with a mean of 100 and standard deviation of 15.

**Table 2:** Association between Reading and television viewing and NIH cognition battery sub-tests and Composite scores.

NIH toolbox score	Daily reading			Daily television viewing		
	β (mean ± SEM)	t-stat	Q-value^a^	β (mean ± SEM)	t-stat	Q-value^a^
Picture Vocabulary (Language)	2·34 ± 0·11	21·99	4·2e-104	−0·56 ± 0·15	−3·75	0·00028
Flanker Inhibitory Control (Executive)	0·83 ± 0·14	6·03	1·7e-09	−0·32 ± 0·13	−2·29	0·022
List Sorting (Working Memory)	1·79 ± 0·18	10·14	5·3e-24	−0·53 ± 0·14	−3·72	0·00028
Card Sorting (Executive)	0·85 ± 0·14	6·01	2·0e-09	−0·59 ± 0·15	−3·79	0·00028
Pattern Comparison (Processing)	0·93 ± 0·23	4·08	4·4e-05	−0·59 ± 0·22	−2·65	0·0089
Picture Sequence (Episodic Memory)	1·35 ± 0·19	7·14	9·8e-13	−0·44 ± 0·16	−2·73	0·0078
Reading Recognition (Language)	2·23 ± 0·095	23·57	9·9e-119	−0·92 ± 0·17	−5·13	9·9e-07
*Fluid Cognition Composite Score*	*1·61 ± 0·15*	*10·63*	*3·2e-26*	*−0·80 ± 0·16*	*−4·83*	*3·5e-06*
*Crystallized Cognition Composite Score*	*2·45 ± 0·089*	*27·58*	*5·4e-160*	*−0·87 ± 0·15*	*−5·51*	*1·9e-07*
***Total Composite Score***	***2·42 ± 0·12***	***20·89***	***2·2e-94***	***−1·00 ± 0·15***	***−6·51***	***8·2e-10***

(a) Q-values are Benjamini and Hochberg FDR adjusted p-value with α = 0·05.

### Associations between reading/television viewing and brain morphology

#### Vertexwise analysis of brain morphology

**Fig. 2:**
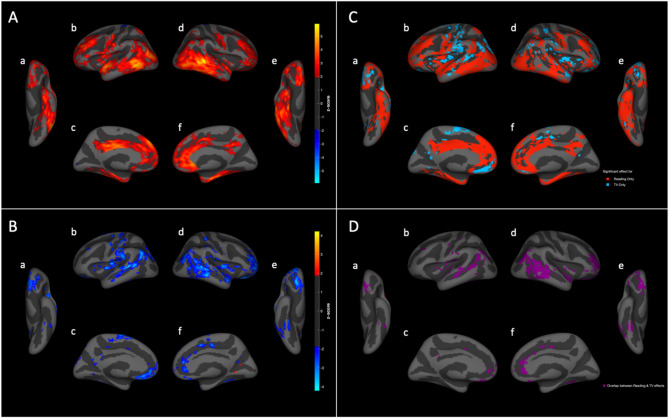
Surface projections of vertexwise associations between cortical area and (**A**) reading and (**B**) television viewing. Heat maps show the beta value z-statistic at each vertex from linear mixed effects models of the relationship between cortical surface area (measured in mm^2^) and daily hours spent (**A**) reading or (**B**) television viewing, both adjusted for subject demographics, family socioeconomic status, genetic ancestry, scanner ID/software version, and for the non-dependent behavior (reading or television viewing hours). Maps are projected onto the inflated cortical surface and thresholded at the z-statistic corresponding to a raw p-value of 0.05. (**C**) Represents the areas of significant effect for only one of reading (red) or television viewing (blue). (**D**) Represents the areas of significant effect overlap between reading and television viewing. Views: a: left ventral; b: left lateral; c: left medial; d: right lateral; e: right ventral; f: right medial.

We used linear mixed effect models to measure vertexwise associations between brain cortical surface area and thickness and daily hours spent reading or television viewing. Three-dimensional projected heat maps (Fig. 2) of vertexwise beta-value z-statistics demonstrate that daily reading is associated with larger cortical area across multiple brain regions, with particularly strong effects along the posterior aspect of the left and right inferior and middle temporal gyri, ventral temporal cortex (fusiform gyrus), left and right mesial parietal lobe (precuneus), and portions of the frontal lobes, specifically the middle and inferior frontal gyri and portions of the cingulate gyrus. Conversely, heat maps showing vertexwise beta-value z-statistics for the effect of daily television viewing indicate that television viewing is associated with smaller cortical area in regions of the frontal, parietal, and temporal lobes, with the strongest associations involving the posterior temporal lobes and temporoparietal junction and orbitofrontal cortex. For clarity, unthresholded versions of these same heat maps, and versions thresholded at a more stringent level of *p* < 0.001 are shown in supplementary eFigs. 4 and 5, respectively. While many regions demonstrating significant associations were specific to reading or TV viewing exposures (Fig. 2C), the lateral temporal and orbitofrontal gyrus regions demonstrated significant associations, but in opposing directions, for reading and TV viewing (Fig. 2D). To assess significance of whole-brain vertexwise results we applied the Multivariate Omnibus Statistical Test (MOSTest)^20,21^, a less conservative estimate than the Bonferroni method that accounts for spatial covariance in vertexwise cortical morphology across the brain, with significance computed by running 10,000 wild bootstrap permutations. The MOSTest p-values for reading and viewing television were < 10^−3^ and 0·027 respectively, confirming significant associations between these exposures and regional cortical surface area, with reading having stronger effects than television viewing. Of note, cortical morphology is characterized by both surface area and thickness; however, in this cohort, morphological associations with reading and television viewing were primarily related to differences in cortical area rather than thickness (eFigure 6 in the Supplement). One factor that is associated with lower reading time and higher TV viewing time, on average, is higher attention difficulties. We therefore also performed the vertexwise analysis of surface area while using the child behavior checklist (CBCL) ADHD t-score as an additional co-regressor. We found no appreciable difference in the size or spatial distribution of surface area effects as a result of this additional co-regressor (eFigure 7).

### Region-of-interest analysis of brain morphology

ROI-based regression of brain morphology on reading and television habits in regions defined by the Desikan-Killiany atlas similarly demonstrates widespread positive and negative associations of cortical gray matter areas with reading and television viewing, respectively (Table 3). Many individual ROIs demonstrated significant positive associations between reading and surface area. While many regions showed negative associations between television viewing and surface area, only a single region – the right inferior parietal lobule – survived correction for multiple comparisons in the ROI analysis.

**Table 3:**
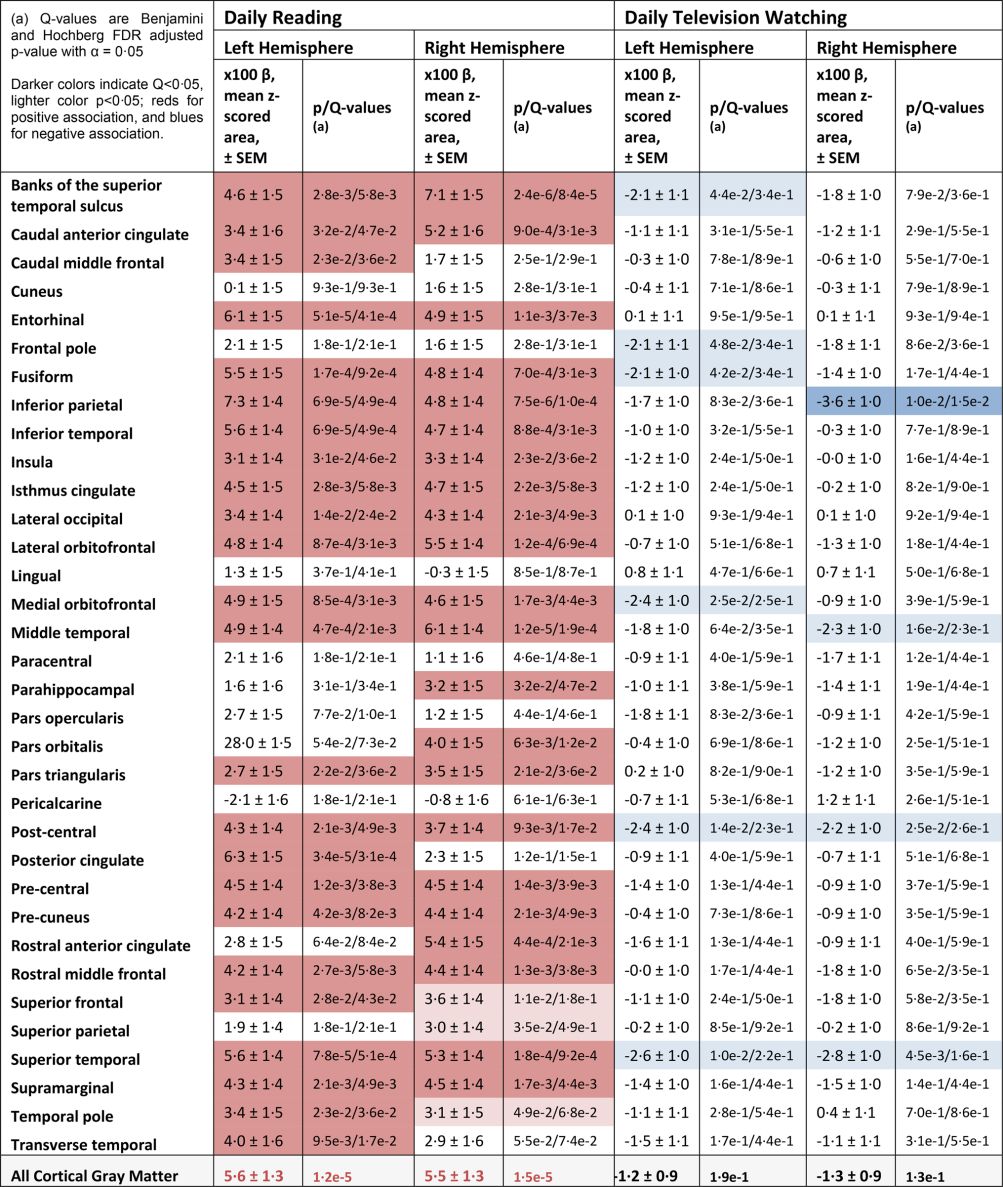
Association between Reading and Television Viewing and Within-ROI cortical area.

### Effect size comparisons

**Fig. 3:**
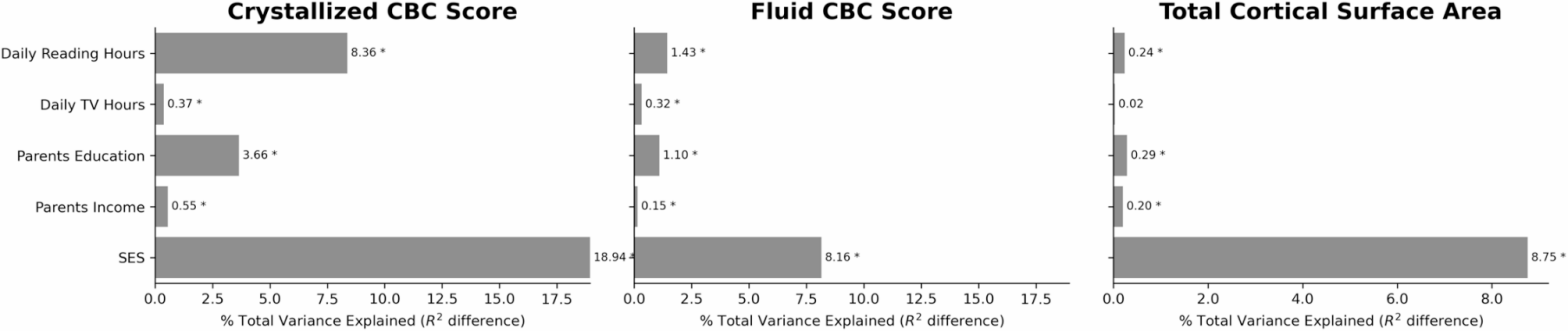
Variance explained by various factors for mixed effects models of NIH Toolbox Cognitive Battery Composite (CBC) Scores (left and middle) and total cortical surface area (right). Effect sizes of individual factors were estimated by calculating the difference in total variance (R^2^) explained by a model that included all covariates compared to a reduced model that lacked the factor of interest (or combination of factors for “SES”). SES = Household Income, Parents Education, Parents Marital Status, Ethnicity, and Genetic Ancestry. *Indicates significance at *p* < 0.01 level.

Using mixed effects models, we measured the unique contributions of various exposures to crystallized and fluid CBC scores and to total cortical surface area. Specifically, the change in percent variance explained in the dependent variable when including or excluding an exposure of interest gives a measure of its unique effect size. Across the population, time spent reading and television viewing accounted for 8.4% and 0.4% of the total variance in crystallized cognition, respectively, and 1.4% and 0.3% of the total variance in fluid cognition (Fig. 3). These effect sizes were similar to the effect size of individual socioeconomic (SES) factors, such as parental education level or household income, although the combined effects of all included SES factors (household income, parental education level, parent marital status, ethnicity, and genetic ancestry) accounted for more variance than either reading or TV viewing.

As expected, effect sizes for reading and television viewing on total cortical surface area were much smaller, accounting for just 0.2% and 0.02% of the total variance, respectively (Fig. 3), and this result was only statistically significant for reading. Effects for specific individual cortical regions were larger than for total cortical surface area (see Table 3 for comparison of beta values). Again, effect sizes for reading were comparable to individual SES factors but smaller than the combined effects of all included SES factors (Fig. 3).

As noted above, because of the inverse relationship between reading and television viewing (eFigure 2) effect size analyses were based on models that included the other factor as a covariate. However, effect sizes (percent total variance explained) were similar when the opposing behavior was not included as a covariate in our mixed effects models (eFigure 8).

## Discussion

Reading books and viewing television are common human behaviors that are colloquially thought to influence neurodevelopmental outcomes, however these associations have never been interrogated on a large scale. Across over eight thousand early adolescents representing a diverse sample of the US population, we found that pleasurable reading and television viewing habits were associated with opposing associations on neurocognitive performance and cortical surface area, although with markedly different effect sizes. Individuals who spend more time reading showed higher neurocognitive performance and a widespread pattern of positive associations with cortical surface area including the bilateral temporal lobes, cingulate gyrus, and dorsolateral and inferior frontal lobes. Individuals who spend more time viewing television showed lower neurocognitive performance and a pattern of negative associations with cortical surface area, most prominently involving the lateral temporal lobes, temporoparietal junction, and orbitofrontal cortex. These associations remained after controlling for participant demographics, family socioeconomic status, genetic ancestry, and MRI scanner equipment effects. The magnitude of reading associations (per unit time) were much larger than those of television viewing.

With respect to NIH Toolbox assessments of neurocognition, reading had its largest associations with picture vocabulary, reading recognition, and crystallized performance, noting that this positive relationship between reading and neurocognition held true only up to 4 h of reading per day. Meanwhile, television viewing had smaller negative associations across all NIH Toolbox scores. Overall, associations per hour of activity were larger for reading than for television viewing in all domains, and the unique variance in cognitive scores accounted for by reading was much larger than that accounted for by television viewing. For brain measures, vertex-wise patterns of associations with cortical surface area were almost all positive for reading and almost all negative for television viewing. Brain regions with positive reading associations are similar to those that differ between good and poor readers^7^, including ventral/posterior temporal cortex and inferior frontal cortex, important nodes within the neural reading circuitry of the dominant hemisphere. Again, overall effect sizes and significance for brain associations were greater for reading than television viewing, reflected in the multivariate whole-brain MOSTest p-values of < 10^−3^ and 0.027, respectively. Indeed, in the atlas-based ROI analyses, only the right inferior parietal lobule showed a significant negative association between cortical surface area and television viewing.

The neural circuitry underlying reading is becoming better understood^7^. For example, white matter microstructure^8^ and global brain network connectivity^9^ have been associated with children’s reading skill, and reading interventions with changes in white matter connectivity^23^, which could in turn affect cortical morphology. However, recent work has highlighted the importance of studying such brain-wide associations in large datasets like ABCD^11^ to yield reliable and reproducible results. Here we leveraged a cohort of over 8,000 youth studied with harmonized behavioral and imaging protocols to show robust, monotonic, opposing associations between reading and both cortical structure and cognitive performance. Time spent reading for pleasure accounted for 8% of the total variance in crystallized cognitive performance, which emphasizes learned knowledge and experience and is heavily influenced by language abilities. Indeed, there is strong correspondence between regions positively associated with time spent reading in this study and regions associated with crystallized cognition in a recent study that examined whole-brain associations between NIH Toolbox cognitive performance and regional cortical morphology in the ABCD cohort^24^. However, in contrast to those results, our results appear entirely mediated by cortical surface area – not thickness – and differ with respect to the left posterior temporal lobe, where surface area shows strong positive associations with time spent reading but not with crystallized composite scores. This region has particularly strong structural and functional connections to the visual word form area (VWFA) of the fusiform gyrus^25^, which is implicated as the gateway through which information about written letters and words reaches higher-order language areas^26^ and may be particularly influenced by reading experience^6^.

Screen time, and in particular television viewing, is increasing^10^ and has been proposed to have detrimental effects on health and cognitive development at various ages^27^. For example, excessive screen time at 2–3 years is associated with poorer performance on developmental tests at 36 months^3^ and has heterogeneous effects on brain structure, psychopathology, and cognitive performance in early adolescents^4^. School age children who meet recommendations for screen time and sleep duration have higher global cognition^28^. Although our results provide some support for the deleterious associations between television viewing, cognitive performance, and brain morphology in developing youth, the magnitude of our results were comparatively small, perhaps reflecting the effects of controlling for confounding variables in the current study.

At the whole-brain level, the size of the relationships between reading or television viewing and brain morphology are small, accounting for just 0.24% and 0.02% of the variance in total cortical surface area, respectively. Such small effects are not surprising given the regionally distributed profile of effects across the cortex at the vertexwise level and the myriad of factors that influence brain morphology. Indeed, the effect size for reading is comparable to that of individual sociodemographic factors such as parental education and household income in the current study, to previously reported behavior-brain associations in ABCD and other large population imaging studies^29,30^, and to medication effects in many large clinical studies, which often account for less than 1% of the variance in the dependent measure^29^. For cognitive performance the unique variance explained by these behaviors was higher, up to 8.4% for the effect of time spent reading on crystallized composite scores.

### Limitations

It is natural to speculate about the causal implications of these results – that reading and television viewing have opposing effects on neuroplasticity that in turn influence neurocognitive development – but we caution against such overinterpretation. First, this is an observational study from which we cannot make causal inferences. Second, our analyses controlled for many confounding factors– including genetic ancestry and sociodemographic factors – but it is possible that our results are influenced by other, unaccounted-for factors. Third, collinearity among some of the modeled factors (eFigure 1 in Supplement) makes it difficult to confidently estimate their independent effects. Nevertheless, each factor contributed additively to the variance explained by our models of cognitive performance and brain morphology, suggesting that they contribute independent predictive power.

Reading and television represent only two activities in which early adolescents engage. However, they have unique significance amongst the behaviors tracked in the ABCD study as they are two common, relatively passive, visually-driven activities that have been both colloquially and academically thought to have opposing associations on neurocognitive development and performance. Other activities and environmental exposures are also likely to be associated with cognitive performance and structural brain measures but are not directly evaluated in this study. Indeed, other factors such as attention difficulties are correlated with certain exposures including reading and television, and the extent to which all possible covariates mediate or explain the results cannot be fully evaluated in this study. Further, the specific content of television programs or reading material is not included as a measure in this study and likely varies across individuals. Finally, the main exposures in the study were acquired via either self- or caregiver-reported surveys and may be subject to response biases and reporting inaccuracy. For example, the daily reading survey had 49 outliers reporting more than 8 h of reading per day, which were excluded on the basis of being seemingly unrealistic behaviors far above the mean. Furthermore, amongst the respondents that were included in the analysis, those with the highest number of daily reading hours (4 to 8) did not show the same linear association with cognitive performance as respondents in the 0 to 4 h/day range. This difference raises the possibility that a subset of the upper range of included reading hours may also be inaccurate, although this set of participants constitutes a relatively small minority of the overall dataset.

## Conclusions

In summary, in this large demographically diverse population of young adolescents, time spent reading and television viewing have largely opposing associations with neurocognitive performance and with cortical brain surface area, noting the much larger magnitude of associations with reading. Although the causal relationships between reading, television viewing, cognition, and brain structure cannot be determined from a cross-sectional study, these findings suggest that regular reading is associated with higher cognitive function and regionally selective cortical area expansion, while television viewing has much smaller opposing associations with these same processes involving different cortical regions. Controlling for a range of potential confounding factors including sociodemographic and genetic ancestry effects, our results suggest that the positive associations of reading on both cognitive scores and brain morphology are much greater in magnitude than the negative associations of TV viewing, and similar in size to the effects of individual socioeconomic factors, such as parental income and education.

## Electronic supplementary material

Below is the link to the electronic supplementary material.

Supplementary Material 1

## Data Availability

The current study is based on imaging, test results, and survey data from participants included in the National Institutes of Mental Health Data Archive (NDA) public release of the baseline data from all children enrolled in the ABCD study (NDA Release 3.0: https://dx.doi.org/10.15154/1460410).
