# Supplementary material for: Neurocognitive and brain structure correlates of reading and television habits in early adolescence
Source: Sci Rep. 2025 Feb 20;15:6235. doi: 10.1038/s41598-025-88398-2 (PMC11842790; doi:10.1038/s41598-025-88398-2)
Supplement: Supplementary file 1 — Supplementary Material 1 [file 41598_2025_88398_MOESM1_ESM.docx]

Supplementary Materials


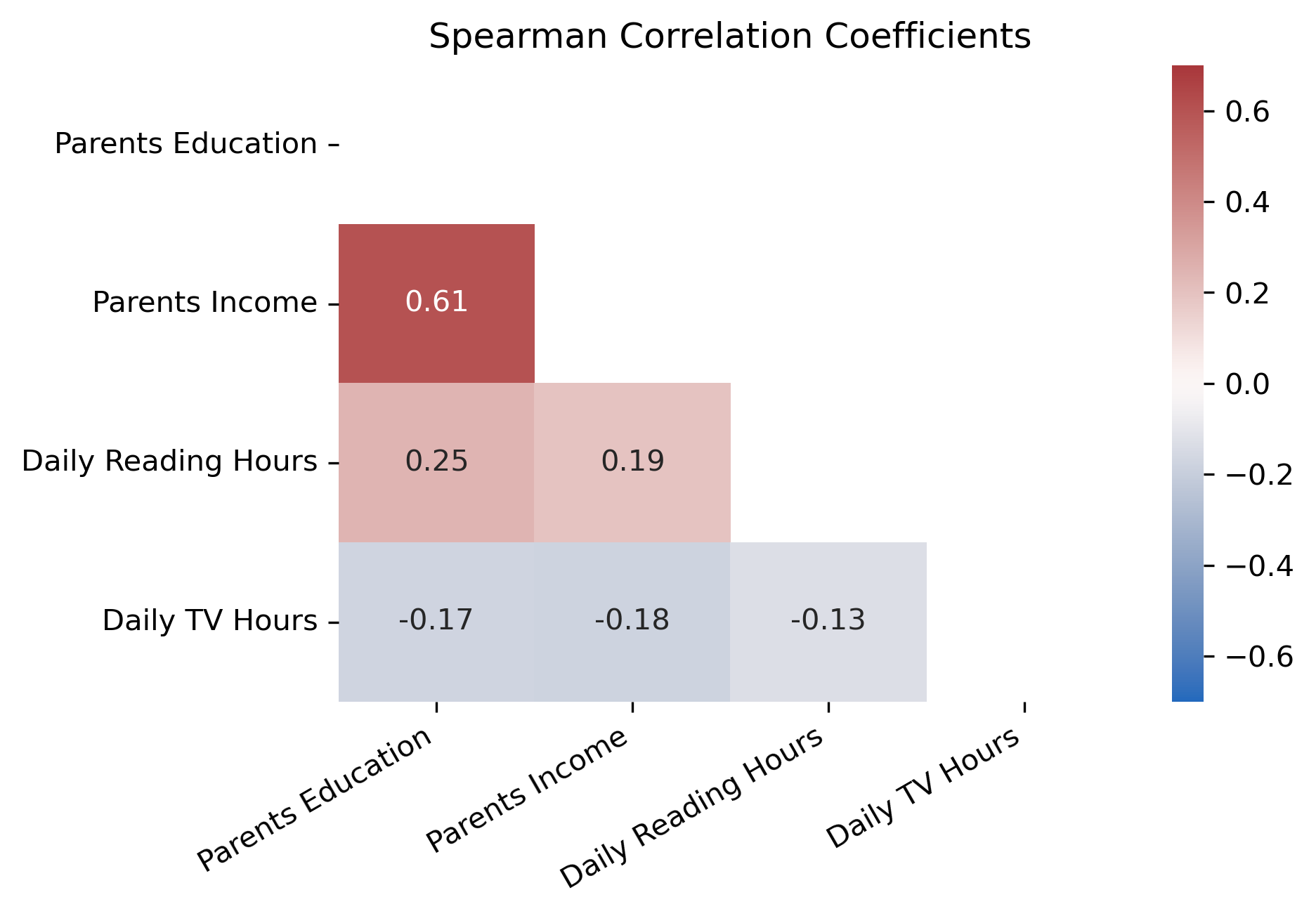


**eFigure 1. Spearman correlation matrix between parents' education, income, and children’s daily pleasurable reading hours and television viewing hours.**

All correlations were significant (p-values < 1e-30).

**eFigure 2. Boxplots comparing reported hours spent daily engaged in pleasurable reading to hours spent daily engaged in television viewing.**


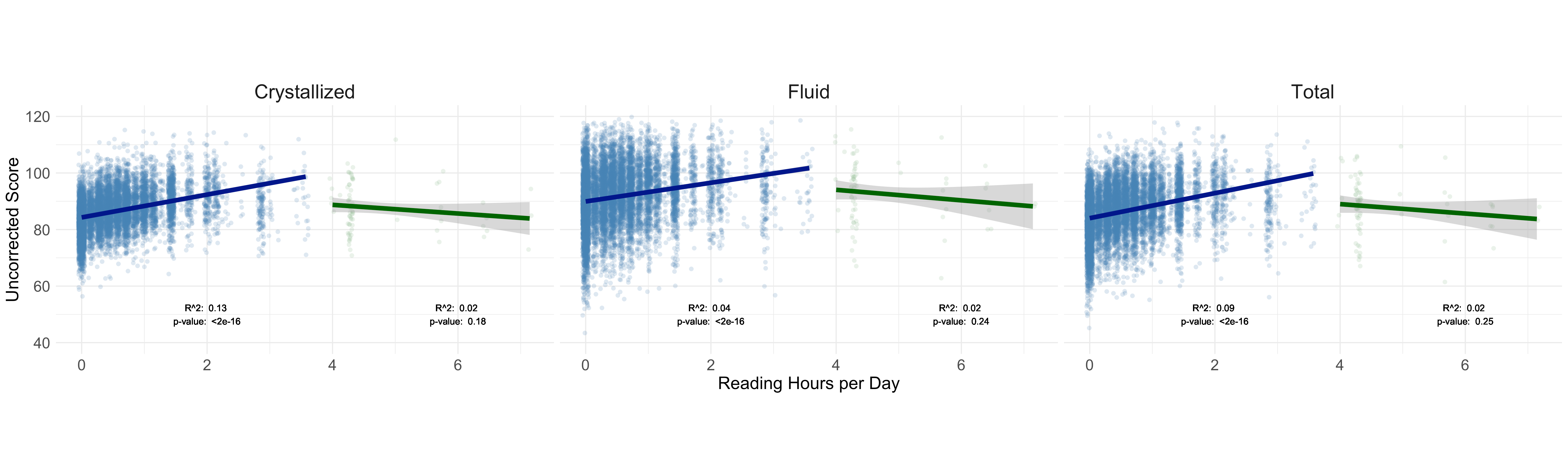


**eFigure 3. NIH Cognition Battery Composite (CBC) Scores vs. Daily Reading Time**. Each value was colored based on the reading time threshold of 4 hours per day (<4h blue, ≥4h green), and the best fit line along with standard error, R^2^, and associated p-value are printed.


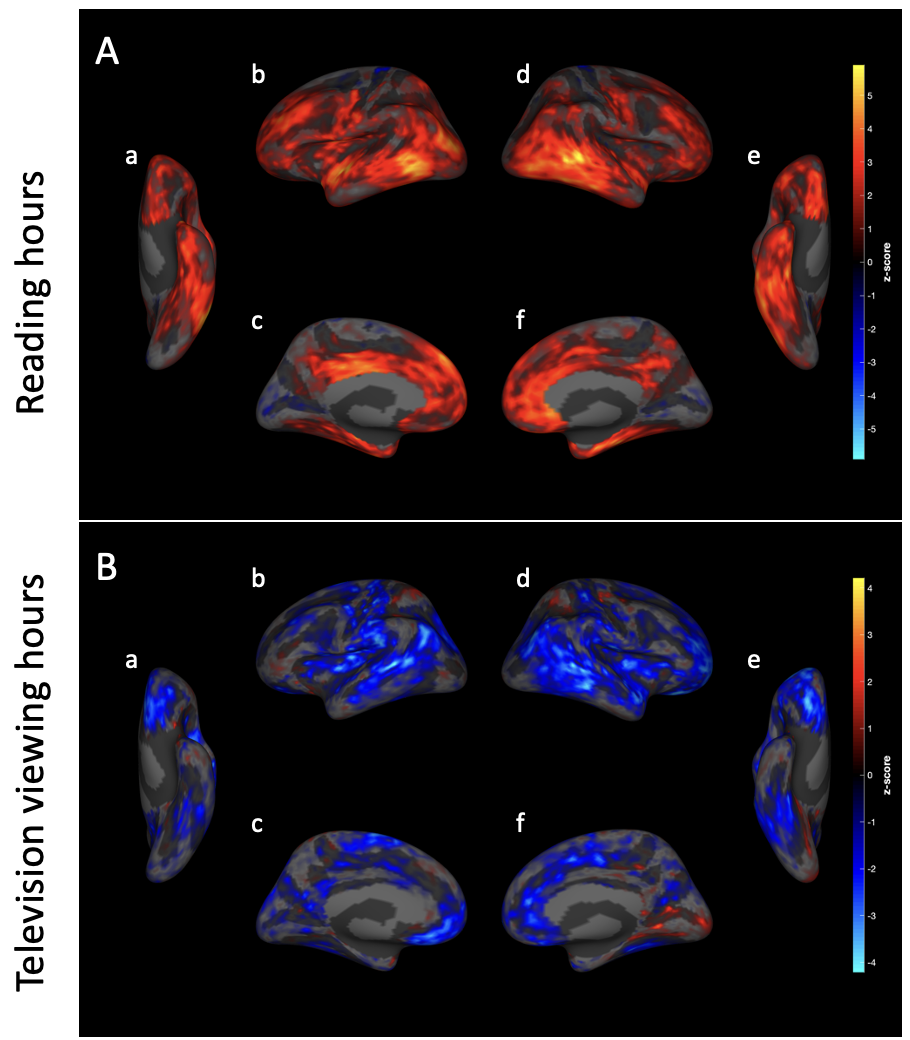


**eFigure 4. Unthresholded surface projections of vertexwise associations between cortical surface area and (A) reading and (B) television viewing.**

Heat maps show the beta value z-statistic at each vertex from linear mixed effects models of the relationship between cortical surface area (measured in mm2) and daily hours spent (A) reading or (B) television viewing, both adjusted for subject demographics, family socioeconomic status, genetic ancestry, scanner ID/software version, and for the non-dependent behavior (reading or television viewing hours). Maps are projected onto the inflated cortical surface and un-thresholded.

**Views:** a: left ventral; b: left lateral; c: left medial; d: right lateral; e: right ventral; f: right medial


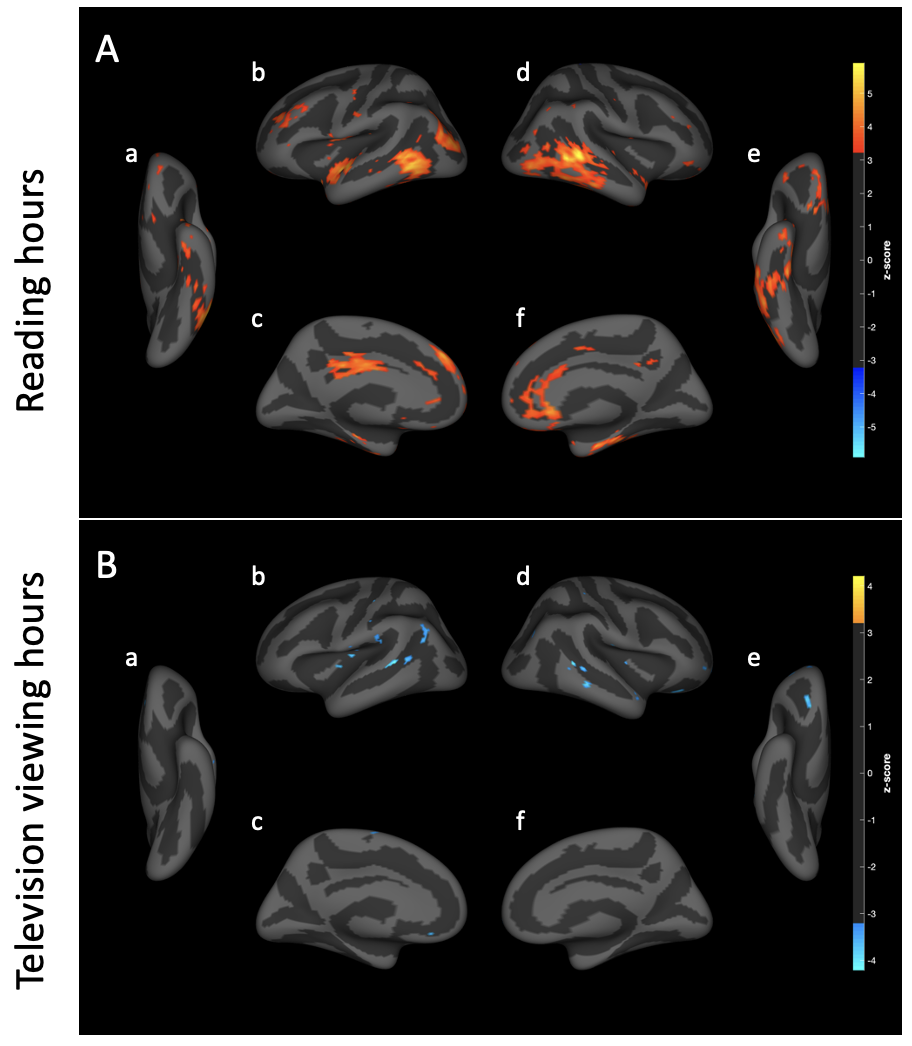


**eFigure 5. Surface projections of vertexwise associations between cortical surface area and (A) reading and (B) television viewing, thresholded at p<0.001.**

Heat maps show the beta value z-statistic at each vertex from linear mixed effects models of the relationship between cortical surface area (measured in mm^2^) and daily hours spent (A) reading or (B) television viewing, both adjusted for subject demographics, family socioeconomic status, genetic ancestry, scanner ID/software version, and for the non-dependent behavior (reading or television viewing hours). Maps are projected onto the inflated cortical surface and thresholded at a p-value of 0.001.

**Views:** a: left ventral; b: left lateral; c: left medial; d: right lateral; e: right ventral; f: right medial


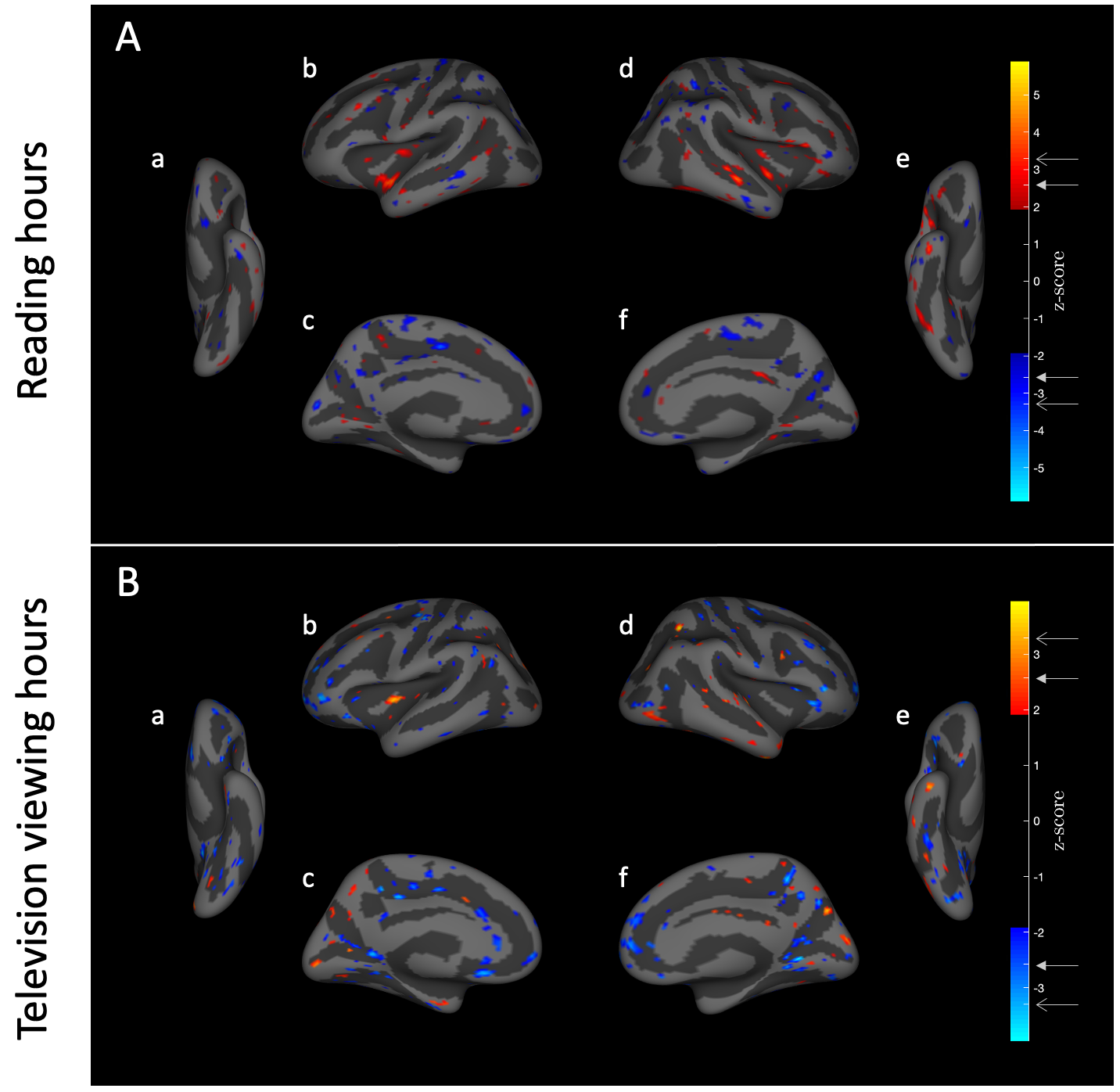


**eFigure 6. Surface projections of vertexwise associations between cortical thickness and (A) reading and (B) television viewing.**

Heat maps show the beta value z-statistic at each vertex from linear mixed effects models of the relationship between cortical thickness (measured in mm) and daily hours spent (A) reading or (B) television viewing, adjusted for subject demographics, family socioeconomic status, genetic ancestry, scanner ID/software version, and for the non-dependent behavior (reading or television viewing hours). Maps are projected onto the inflated cortical surface and thresholded at the z-statistic corresponding to a raw p-value of 0.05.

**Views:** a: left ventral; b: left lateral; c: left medial; d: right lateral; e: right ventral; f: right medial


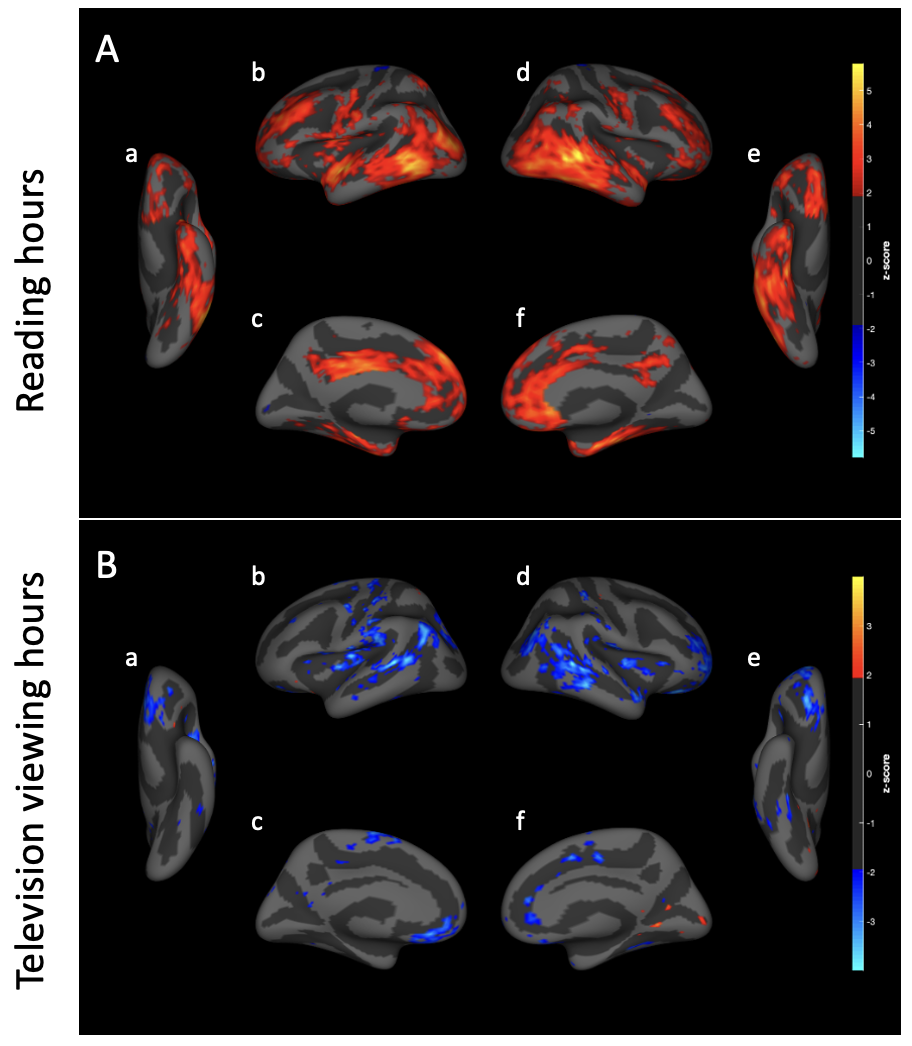


**eFigure 7. Surface projections of vertexwise associations between cortical area and (A) reading and (B) television viewing, when including ADHD t-score as a co-regressor.**

Heat maps show the beta value z-statistic at each vertex from linear mixed effects models of the relationship between cortical surface area (measured in mm^2^) and daily hours spent (A) reading or (B) television viewing, both **adjusted for ADHD t-score**, subject demographics, family socioeconomic status, genetic ancestry, scanner ID/software version, and for the non-dependent behavior (reading or television viewing hours). Maps are projected onto the inflated cortical surface and thresholded at the z-statistic corresponding to a raw p-value of 0.05.

**Views**: a: left ventral; b: left lateral; c: left medial; d: right lateral; e: right ventral; f: right medial.


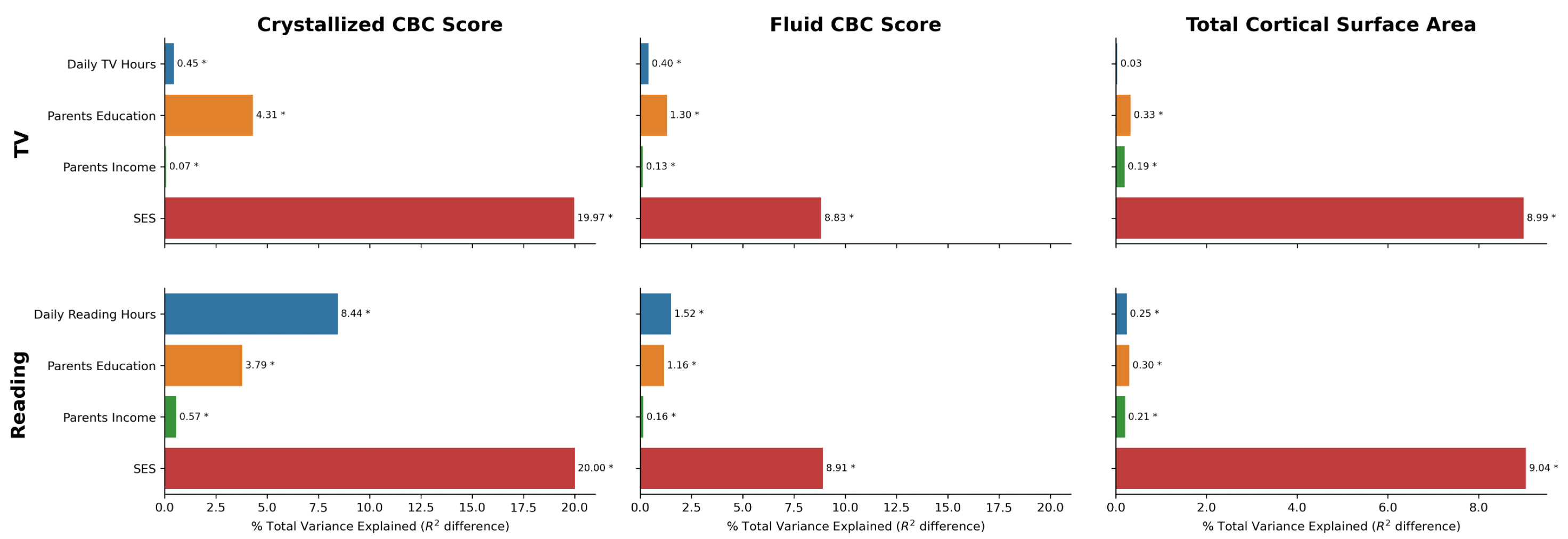


**eFigure 8. Variance explained by various factors for mixed effects models of the effect of TV (first row) or Reading (second row) on NIH Toolbox Cognitive Battery Composite (CBC) Scores (left and middle) and total cortical surface area (right).**

In contrast to Figure 3 in the main text, these models included only reading or television viewing (i.e., they did not include the other behavior as a covariate). Effect sizes of individual factors were estimated by calculating the difference in total variance (R^2^) explained by a model that included all covariates compared to a reduced model that lacked the factor of interest (or combination of factors for “SES”).

SES = Household Income, Parents Education, Parents Marital Status, Ethnicity, and Genetic Ancestry.

* indicates significance at p<0·01 level.
